# Current Status of Biology–Biotechnic, Agronomic, and Biological Control of *Rhynchophorus ferrugineus*: A Review

**DOI:** 10.3390/insects15120955

**Published:** 2024-11-30

**Authors:** Waqas Wakil, Maria C. Boukouvala, Nickolas G. Kavallieratos, Constantin S. Filintas, Nikoleta Eleftheriadou, Muhammad Usman Ghazanfar, Muhammad Yasin, Mirza Abdul Qayyum, Pasco B. Avery

**Affiliations:** 1 Department of Entomology, University of Agriculture, Faisalabad 38040, Pakistan; yasin_1876@yahoo.com; 2 Senckenberg German Entomological Institute, D-15374 Müncheberg, Germany; 3 Laboratory of Agricultural Zoology and Entomology, Department of Crop Science, Agricultural University of Athens, 75 Iera Odos Str., 11855 Athens, Greece; mbouk@aua.gr (M.C.B.); p1172219@aua.gr (C.S.F.); nikolelef@aua.gr (N.E.); 4 Department of Plant Pathology, University of Sargodha, Sargodha 40100, Pakistan; usmanghazanfar@uos.edu.pk; 5 Department of Entomology, Faculty of Agriculture and Environment, The Islamia University of Bahawalpur, Bahawalpur 63100, Pakistan; 6 Institute of Plant Protection, Muhammad Nawaz Shareef (MNS) University of Agriculture, Multan 60000, Pakistan; qayyum.mirza@mnsuam.edu.pk; 7 Department of Entomology and Nematology, Institute for Agricultural Sciences, Indian River and Research Center, University of Florida, Fort Pierce, FL 34945, USA; pbavery@ufl.edu

**Keywords:** red palm weevil, life history, distribution, semiochemicals, natural enemies, microbial control agents, host plants

## Abstract

*Rhynchophorus ferrugineus* (Olivier) (Coleoptera: Curculionidae), the red palm weevil (RPW), is a concealed voracious pest of different ornamental and economically important palm species, particularly the date palm. It can cause huge losses in ornamental and commercial palm plantations. RPW has spread rapidly from its original distribution in Southeast Asia to date palm-growing countries worldwide. It is now established in more than 50% of date palm-growing countries and 15% of the coconut-growing countries globally. To prevent further expansion of this pest, many countries have implemented strict legislative and quarantine measures surrounding the export of the palms plant genetic resources from RPW-infested countries. This review focuses on the general biology (life history, development), host range, geographical distribution, and management on the basis of biotechnic methods, farming practices, natural enemies, and important microbial control agents.

## Simple Summary

*Rhynchophorus ferrugineus* is a noxious curculionid species found in date palm and coconut plantations worldwide. Its overall cryptic nature inhibits early detection of infestation symptoms and allows for its rapid expansion. Control methods in plantations usually include single-mode broad-spectrum chemical insecticides to both prevent and mitigate infestations. However, ecological concerns about hazards of both ecosystem and public health, call for safer and more sustainable solutions, including attractants, agronomic approaches, natural enemies and entomopathogenic organisms. This review highlights the published information on the biological traits, host plant spectrum, and management options such as biotechnic and biological control methods like the application of microbial organisms.

## 1. Introduction

Insect invasion is highly damaging to natural, urban, and agricultural areas, causing not only ecological damage but also economic losses of several million dollars (USD) per year related to the cost of control efforts to reduce populations to lower levels [1,2,3]. The red palm weevil (RPW), *Rhynchophorus ferrugineus* (Olivier) (Coleoptera: Curculionidae) is a serious invasive pest of high economic importance that invades the tissues of several palm species globally [4,5]. The historical evidence showed that this pest originated from South and Southeast Asia [6] and infested coconut [7], and also date palms in Mesopotamia (Iraq) [8] but was not recognized as having prominent pest status on date palms until the mid-1980s in the Middle Eastern region [9,10]. RPW mainly infested *Phoenix canariensis*, in the Mediterranean Basin of the Canary Islands, slowly during the mid-1990s and very quickly since 2004; consequently, a mass-eradication campaign was carried out to overcome this pest [11]. Most individuals of this pest are short-distance flyers covering < 500 m, and this might be the reason for the appearance of hot spots [12]. The invasion potential of RPW is due to the increased fecundity of females, which can produce multiple generations per year as a multivoltine species. Its development takes place inside the host palm delaying early detection of this pest, and the high flight capacity of adults can cover long distances [13,14,15,16,17]. However, a major factor in its spread has been the movement of infested palm material into uninfested areas [16]. 

**Figure 1:**
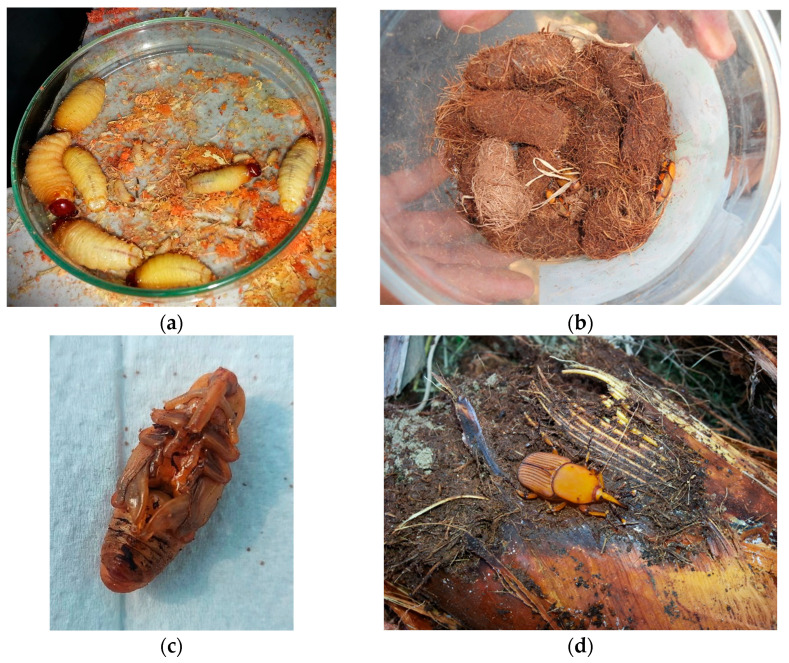
*Rhynchophorus ferrugineus*: (**a**) larvae, (**b**) cocoons created by pupating larvae, (**c**) pupa, (**d**) adult male on date palm tree.

RPW has emerged as the largest weevil within the fauna of Europe and North Africa [18]. The physical dimensions of RPW vary significantly, ranging from 1.5 to 4.0 cm in length (from the tip of the rostrum to the end of the abdomen) and from 0.7 to 1.5 cm in width [18], with males being approximately 10–15% smaller and lighter than females [19]. Typically, the length of individuals in the Mediterranean region is about 3 cm [18]. Adults display variable coloration, often characterized by a reddish-brown thorax with varying degrees of distinct black mottling, as well as black and orange stripes on the elytra (Figure 1d) [20]. Male weevils can be identified from females by the presence of a tuft of reddish-brown hairs along the dorsal side of the snout which is shorter and thicker compared to that of females [18]. Additionally, the front tibiae show sexual dimorphism, with males having a comb-like brush of tightly packed long hairs, while females have only sparse hairs [18,21]. Adults have robust wings that allow them to perform extended flights [11]. Several studies using the flight mill technique have shown the remarkable ability of RPW to fly long distances. For instance, Hoddle et al. [22] recorded that 3 of 192 RPW individuals tested flew a maximum distance of 60–67 km in 24 h, whereas the maximum flight distance of RPW in 6 hours was 23.6 km [13]. The eggs are elongated with a length of 0.25 cm, whitish color, and smooth chorion [18]. The larvae are apodous, yellowish or whitish in color, up to 50 mm long. The head color ranges from russet-red to brown, with strong mandibles (Figure 1a) [23,24]. Full-bodied larvae create a cocoon composed of palm fibers and saliva (Figure 1b,c) where they pupate [25]. Pupae change color from cream to brown, and the average size is approx. 4.5 cm [21].

Palm mortality occurs as a result of larvae feeding internally [14,26,27,28]. The whole life cycle of larvae remains cryptic inside the palm, posing challenges for the early detection of infestations [26,27]. In addition, the larvae are protected from external factors that can cause their death, such as insect pathogens, natural enemies, and insecticides [29]. The feeding activity of larvae over 1 to 2 years can lead to the death of infested palm trees by causing severe damage to apical growing areas and/or resulting in trunk collapse [27,30,31].

**Figure 2:**
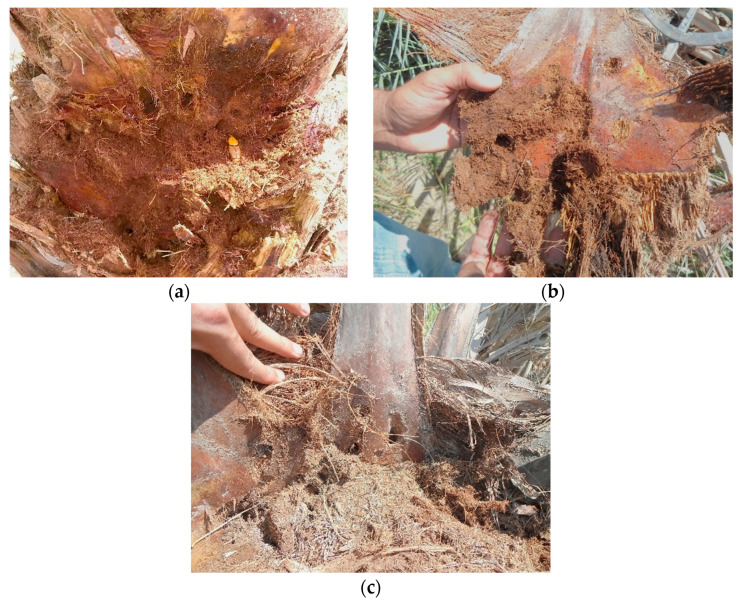
Feeding damage symptoms in date palm trees caused by *Rhynchophorus ferrugineus*: (**a**) severe internal damage at the base of the palm trunk, with visible tissue decay and tunneling caused by larval feeding, (**b**) cross-section showing extensive frass accumulation and damage to the internal fibers of the fronds, and (**c**) frond base exhibiting boreholes and internal damage, indicating the presence of larval tunnels.

RPW adults are usually attracted to unhealthy and damaged plants from pruning or chainsaw wounds [23]. Fermenting sap of injured trees releases a chemical that attracts RPW to palms [32]. Also, due to the gregarious behavior of the RPW males [33], a pheromone comprising of two compounds, ferrugineol (4-methyl-5-nonanol) and ferrugineone (4-methyl-5-nonanone), induces aggregation [34]. Infestations in *Phoenix dactylifera* L. (Arecales: Arecaceae) are often recorded in younger palms (<20 years old plants) at approximately 1 m on the trunk, above the soil [18,23,35,36]. Detection of early infestation is challenging as symptoms typically manifest long after the infestation has occurred. Consequently, significant feeding damage inflicted by numerous larvae usually leads to the demise of the palm before symptoms become apparent [37]. Detailed descriptions of symptoms of feeding damage on date palm trees have been provided in the literature [35,37]. Damaged trees include the following symptoms: (i) tunnels at the base of the petiole (Figure 2b,c) and along the trunk (Figure 2a), (ii) audible gnawing sounds caused by grub feeding, (iii) discharge of brownish viscous liquid and chewed fibers emerging from small holes in the stem and crown, (iv) the occurrence of frass (chewed plant tissues), emitting a characteristic fermented odor around the tunnel openings, and (v) the presence of discarded empty pupal cases and dead adults around an infested palm [14,35,37,38,39,40]. Therefore, possessing a comprehensive understanding of the diverse active feeding symptoms exhibited by infested palms is crucial for promptly identifying RPW infestation [40].

Various methods are available or have been tried for the early detection of RPW: (1) Detection of infested palms by visual inspection is still a common practice [37]. Regular inspection of susceptible palm trees (mostly < 20 years) [14,41] at 1.5-month intervals is a useful tool to restrict the accumulation of RPW in the field since infestations are located prior to adult emergence, which takes approximately 45 days from egg to adult [13]. (2) Training dogs to smell specific chemical signals emitted by RPW-infested date palms is a potential method for early detection [42,43]. For instance, Nakash et al. [44] validated the capability of Golden Retriever dogs to effectively detect the oozing secretion extracted from RPW-infested date palms by sniffing. However, Soroker et al. [43] suggested that the use of dogs for RPW detection purposes could also be appropriate for palm inspection at quarantine facilities, nurseries, and ports of entry. (3) Acoustic methods have proven effective for the detection of both adult and larval stages of RPW within palm trees [45,46,47]. Several researchers have extensively investigated the acoustic activity of RPW, determining that the sound emitted by the weevil can be distinguished and separated from ambient noises and other insect sounds [48,49,50,51]. Pinhas et al. [52] devised a mathematical approach for the automated detection of RPW acoustic activity in plant offshoots. The Laar WD 60 Pro CSC measuring device was regarded as the most optimal and straightforward choice for detecting subtle sound vibrations generated by RPW activity during the initial stages of infestation [32,53]. The detection of RPW during the initial stages of infestation using a bioacoustic sensor developed by Rach et al. [54], proved to be very effective, achieving 90% success, despite the ambient sounds. Martin et al. [51] found that the sound spectrum of RPW larvae remains consistent during biting and chewing actions but varies during the insect’s locomotion. The IoTree Smart acoustic sensor was assessed for its ability to detect sound signals emitted by RPW larvae inside oil palm and coconut trees in Malaysia [55]. This sensor detected the presence of the pest in both plant species. Using data mining, RPW infestations can be predicted with an accuracy up to 93%; however, temperature and tree trunkcircumference are the most important features for this prediction [56].

Since there is a lack of review information dealing with the RPW biology from a biotechnic/biological control point of view, we focused this review on recent information obtained from the available global published literature on the following topics: affected plants, global distribution, life history traits, and different biological management methods (i.e., semiochemicals, agronomic practices, natural enemies, entomopathogens). 

## 2. Host Range

RPW is a polyphagous pest that has been recorded to attack more than 40 palm species worldwide [14,46,47,48,49,50,51,52,53,54,55,57,58,59,60], causing widespread devastation of date palms grown in the Canary Islands, the Mediterranean, Middle East and North Africa [14,27,61]. Table 1 presents the plant species affected by RPW.

**Table 1:** List of host plants of *Rhynchophorus ferrugineus*.

Host Species	Order	Family	References
*Areca catechu* L.	Arecales	Arecaceae	[62,63,64]
*Arenga pinnata* (Wurmb) Merr.	Arecales	Arecaceae	[26,37,63,65,66]
*Bismarckia nobilis* Hildebr. & H.Wendl.	Arecales	Arecaceae	[37]
*Borassus flabellifer* L.	Arecales	Arecaceae	[37,65,66]
*Brahea armata* S.Watson	Arecales	Arecaceae	[37,66]
*Butia capitata* (Mart.) Becc.	Arecales	Arecaceae	[37,66]
*Calamus merrillii* Becc.	Arecales	Arecaceae	[37,66]
*Caryota cumingii* Lodd. ex Mart.	Arecales	Arecaceae	[37,63,66]
*Caryota maxima* Blume ex Mart.	Arecales	Arecaceae	[37,63,66]
*Chamaerops humilis* L.	Arecales	Arecaceae	[37,57,66,67,68]
*Cocos nucifera* L.	Arecales	Arecaceae	[27,37,63,66,69,70]
*Corypha umbraculifera* L.	Arecales	Arecaceae	[65]
*Corypha utan* Lamk.	Arecales	Arecaceae	[37,63]
*Dictyosperma album* (Bory) H. Wendl. & Drude ex Scheffer	Arecales	Arecaceae	[37,71]
*Elaeis guineensis* Jacq.	Arecales	Arecaceae	[26,27,65,66,72,73]
*Howea forsteriana* Becc.	Arecales	Arecaceae	[37,66]
*Jubaea chilensis* (Molina) Baill.	Arecales	Arecaceae	[37,66]
*Livistona chinensis* (Jacq.) R.Br. ex Mart.	Arecales	Arecaceae	[26,65,73]
*Livistona decora* (W. Bull) Dowe	Arecales	Arecaceae	[26,37]
*Livistona saribus* (Lour.) Merr. ex A.Chev.	Arecales	Arecaceae	[37]
*Metroxylon sagu* Rottb.	Arecales	Arecaceae	[4,26,37,65,66]
*Oncosperma horridum* (Griff.) Scheff	Arecales	Arecaceae	[37]
*Oncosperma tigillarium* (Jack) Ridl.	Arecales	Arecaceae	[37]
*Phoenix canariensis* Chabaud	Arecales	Arecaceae	[11,18,26,37,66,74,75]
*Phoenix dactylifera* L.	Arecales	Arecaceae	[18,26,37,65,66,70,74,75,76,77]
*Phoenix sylvestris* (L.) Roxb.	Arecales	Arecaceae	[37,65,75,78]
*Phoenix theophrasti* Greuter	Arecales	Arecaceae	[30,37,66]
*Pritchardia pacifica* Seem. & H.Wendl.	Arecales	Arecaceae	[37]
*Roystonea regia* (Kunth) O.F.Cook	Arecales	Arecaceae	[37]
*Sabal palmetto* (Walt.) Lodd.	Arecales	Arecaceae	[37]
*Saccharum officinarum* L.	Poales	Poaceae	[37]
*Strelitzia nicolai* Regel & Körn.	Zingiberales	Strelitziaceae	[37,79]
*Syagrus romanzoffiana* (Cham.) Glassman	Arecales	Arecaceae	[37,80]
*Trachycarpus fortunei* (Hook.) H. Wendl.	Arecales	Arecaceae	[37,66]
*Washingtonia filifera* (L. Lindl)	Arecales	Arecaceae	[37]
*Washingtonia robusta* H.Wendl.	Arecales	Arecaceae	[37]

## 3. Geographic Distribution

RPW is a destructive pest of the date palm and has been detected in more than 50% of date-producing countries and 15% of coconut-producing countries [14]. It was first identified outside its native range in Japan in 1975 [81]. Originating from Southeast Asia, during the 1980s, RPW was recognized as an important pest of dates in the Middle East region, showing rapid spread to other countries through the trade of infected ornamental plants [14,82,83]. RPW expanded to the Arabian Peninsula and into the eastern Mediterranean and Spain by the 1990s [18]. It has since been found in more distant regions, including the Canary Islands, Madeira, the Caribbean, Taiwan, and China. By the year 2015, RPW had been reported in 18 of the 21 Mediterranean coastal countries [32,37].

According to the database of the European and Mediterranean Plant Protection Organization [37], in Africa, the RPW is present in Djibouti and widespread in Egypt, and in Tunisia with restricted distribution, while in Libya, Mauritania, and Morocco it is present with few occurrences. The severe outbreak of RPW in the oasis regions of the above-mentioned countries may lead to social issues due to religious and cultural significance [84,85,86]. RPWt is found in Central America, Caribbean, present in the Netherlands and Antilles with few occurrences, and has restricted distribution in Aruba and Guadeloupe. In Asia, it is widespread in India and Saudi Arabia; present in Bahrain, Bangladesh, Cambodia, China, Iran, Kuwait, Lebanon, Myanmar, Oman, Pakistan, Philippines, Qatar, Sri Lanka, Syria, Taiwan, Thailand, United Arab Emirates, and Vietnam. It is also present in Israel, Japan, Malaysia, Yemen with restricted distribution, and Iraq, and Jordan with few occurrences. In Europe, RPW is present in Albania, Georgia, and Malta, is transient in Bulgaria, and has restricted distribution in Bosnia and Herzegovina, Croatia, Cyprus, France, Greece, Italy, Montenegro, Portugal, Russia, Spain, and Turkey. Currently, according to the European and Mediterranean Plant Protection Organization (EPPO), RPW has been a quarantine pest in Israel since 2009, in Tunisia since 2012, Morocco and Mexico since 2018, and China since 2021 [37]. In addition, RPW has been categorized in the EPPO A1 list (pests are absent in the region of EPPO) in Brazil and Georgia since 2018, and United Kingdom since 2020, as well as on the EPPO A2 list (pests are present locally in the region of EPPO) in Bahrain since 2003, Jordan since 2013, Turkey since 2016, and Egypt and Iran since 2018.

## 4. Life History

Life history studies of RPW have been conducted by several authors in different countries, such as Iran, Philippines, India, Spain, Myanmar, and Indonesia [29,87]. RPW can mate throughout the day, with males and females participating in multiple matings during their lifetime [37,88,89]. RPW undergoes four developmental stages, namely, egg, larva, pupa, and adult to complete its life cycle. The female oviposits separately elongated shiny creamy-white colored eggs (2.5 mm × 1 mm) into excavated holes of a suitable host material which are eventually covered with a dry secretion [86,90]. The daily egg-laying ability of the female decreases with time [75]. A newly hatched larva measures only 5 mm × 2 mm and weighs about 1 mg, while the final larval instar can reach 5 cm × 2 cm and weigh between 4 and 7 g before pupation (Figure 1a). The final larval instar forms a barrel-shaped cocoon made from palm tree fibers measuring 7 cm × 4 cm, while the pupa inside is 3.5 cm × 1.5 cm [86] (Figure 1b,c). Mating duration ranges from 60 to 300 seconds [89], and females use the sperm from their most recent mating to fertilize their eggs [88]. Females begin oviposition within 1 to 11 days after copulation depending on temperature [91], typically selecting tender parts with small scooped holes in the young palm trees (below the age of 20 years [69]), whereas, in mature trees, egg deposition occurs in exposed plant tissues, wounds, petioles, and injuries resulting from another coleopteran pest, *Oryctes rhinoceros* (L.) (Coleoptera: Scarabaeidae) activity or diseases [14,73]. The number of eggs a female can lay in its lifetime ranges from 3 to 531 (mean = 250) [27,92].

Larval hatch occurs after 4 to 7 days [91]. The newly emerged apodous larvae remain exposed to the external environment for a few hours and then enter the trunk, creating tunnels by chewing the trunk tissues [88] (Figure 2). The larvae’s abdominal muscles occasionally contract, which helps them move further into the trunk [88]. Different studies have revealed considerable variation in larval development periods and number of instars [11]. The duration of the developmental period is influenced by factors such as temperature and feeding host, varying between 24 and 210 days [5,11,88]. Seven larval instars were reported by Esteban-Durán et al. [93] when RPW was reared on sugarcane, while Martín and Cabello, [94] recorded 11–17 instars when reared on the same substrate. As the larvae progress through each subsequent instar, their appetite increases, and they primarily consume the soft tissues that surround the apical meristem. During the final larval instar, they migrate to the periphery to pupate, creating a cocoon composed of chewed fibers [88] (Figure 1). The duration of the pupal period can range from 20 to 25 days [95].

After the adults emerge, they typically stay within the cocoon for a duration of 4 to 17 days, with an average period of 8 days [21], possibly to complete sexual maturation [21]. Regardless of sex, adult individuals live for a period of 2 to 3 months [37]. However, host plants significantly contribute to the wide range of adult lifespan durations, acting as one of the main factors influencing growth periods [96]. Due to overlapping generations of RPW, the pest can be found throughout the year on infested palms. The insect can complete three to four generations within a single palm tree over the course of a year [6]. However, in a study by Salama et al. [97], 21 generations were recorded per year in Egypt. It has been proposed that one pair of RPW could potentially generate over 53 million offspring across four successive generations if control factors are absent [14]. Since there are no complex behaviors required for mating of RPW and due to the similarity of variation in genitalia among the genera, interspecific mating (between *Rhynchophorus* spp.) is possible [36].

## 5. Management

### 5.1. Biotechnic: Semiochemicals/Trapping

The use of traps baited with aggregation pheromones represents a promising method for capturing and killing the RPW [7]. The current management approach for RPW involves aggregation pheromones used for both monitoring and mass trapping [60,98,99,100]. In general, for weevils, semiochemical-based trap systems consist of three main parts: a trap, an aggregation pheromone, and a co-attractant (kairomone) [101]. Under natural conditions, males of RPW secrete the aggregation pheromone consisting of ferrugineol (i.e., 4-methyl-5-nonanol) and ferrugineone (i.e., 4-methyl-5-nonanone) at a 9:1 ratio, with ferrugineol comprising the primary component [102]. The aggregation pheromone instigates synchronized mass attacks of RPW adults against the tree host, which frequently results in the collapse or demise of the palm [102,103]. Males and females are attracted to the aggregation pheromone, with a notable preference for females, which makes it extremely advantageous for the mass trapping method. The sex ratio of captured RPW has been reported to be 1 male: 2 females [14,76,104,105,106]. Furthermore, it has been found that most RPW individuals captured in the traps are young and fertile, which suggests a significant reduction in the local population due to this method [35,107]. Both components of the RPW aggregation pheromone have been produced in a commercial lure called Ferrolure^+^, while the main component ferrugineol is commercially produced with the name Ferrolure [108]. 

Several research efforts have shown that RPW is highly attracted to ferrugineol, especially when a food source is mixed with the pheromone [35,109]. Studies have shown that natural palm baits exhibit weak attractive properties on their own, but can greatly enhance the effectiveness of the RPW aggregation pheromone [9]. In addition, the palm esters ethyl butyrate, ethyl isobutyrate, ethyl acetate, and ethyl propionate are among the volatile compounds released from fermented tissues of various hosts to which RPW responds, as has been determined by electroantenographic (EAG) bioassays [110,111,112]. For instance, RPW captures were doubled when a 1:3 mixture of ethyl acetate and ethanol was used with the aggregation pheromone compared to the pheromone alone [111]. Abdel-Azim et al. [113] reported that the efficacy of Ferrolure^+^ was enhanced with the addition of ethyl acetate. In Table 2 we review in detail the attractants and food baits used to capture RPW. After RPW adults enter the pheromone trap, it is important to prevent their escape by immobilizing or killing them with an insecticide mixed into the bait [27,113,114,115].

**Table 2:** Attractants and food baits used for the capture of *Rhynchophorus ferrugineus*.

Substances	Conditions	Target Plantation	References
^1^ Ferrolure^+^ + food baits (ripe date fruits or date palm stems)	Field	Date palm	[116]
Male aggregation pheromones + food baits	Field	Date palm	[117]
^2^ Ferrugineol + date fruits	Field	Date palm	[76]
Ferrugineol + pineapple fruit	Field	Coconut	[109]
Ferrugineol + sago palm stem			
Ferrugineol + sugarcane stem			
Yellow funnel type trap + Pherodis	Field	Palm trees	[118]
Yellow pitfall trap + Pherodis			
Ferrolure^+^ + ethyl acetate	Field	Canary Palm	[119]
Ethyl acetate	Field	Date palm	[120]
Ferugineol + sugar beet molasses	Field	Date Palm	[101]
Male aggregation pheromones + water or paraffin	Field	Date Palm	[121]
Ferrolure^+^	Field	Date Palm	[122]
Male aggregation pheromone + food baits (sugarcane or pineapple or coconut fruit or oil palm petiole) + ethanol + ethyl acetate +water	Field	Coconut	[123]
Ferrolure^+^	Field	Date Palm	[106]
Ferrugineol + fermenting date fruits+ ethyl acetate	Field	Date Palm	[124]
Ferrolure^+^ + ethyl acetate + different trap colors	Field	Date Palm	[125]
Ferrugineol + ethyl acetate + food bait (sugar cane)	Field	Coconut	[126]
Ferrolure^+^ + date fruits	Field	Date palm	[99]
^3^ Rhylure-700 + date fruits			
Ferrugineol + ethyl acetate	Field	Date Palm	[127]
Pineapple or sugar cane or coconut or oil palm	Field	Date Palm	[128]
Ethyl acetate or ethyl butyrate or ethyl propionate or ethylene glycol			
Ferrugineol + aqueous solution of sugar beet + ethyl acetate + ethyl propionate	Field	Date Palm	[129]
Ferrolure^+^+ sugar beet molasses + ethyl acetate	Field	Canary Island Palms	[130]
Ferrolure^+^ + ethyl acetate + sugarcane sticks	Field	Coconut	[131]
Ferrolure^+^ + ethyl acetate + date fruits	Field	Date Palm	[132]
Ferrugineol + date palm tissue	Field	Date Palm	[133]
Ferrolure^+^ + ethyl acetate + date fodder	Field	Date Palm	[134]
Ferrugineol + ethyl acetate	Field	Date palm	[135]
Ferrolure^+^ + date fodder	Field	Date Palm	[136]
^1^ Rhyfer 700	Field	Date Palm	[137]
^3^ Pherocon RPW lure			
^1^ Ferrugitom 700			
^1^ Weevil lure			
Ferrolure^+^			
Ferrugineol + food baits (date fruits or palm fronds)	Field	Date Palm	[138]
Ferrugineol	Laboratory, Field	Coconut	[139]
Ferrugineol + fermented date fruits solution	Laboratory, Field	Date Palm	[35]

^1^ 4-Methyl-5-nonanol (9 parts) + 4-methyl-5-nonanol (1 part). ^2^ 4-Methyl-5-nonanol. ^3^ 4-Methyl-5-nonanol (31.5%) + 4-Methyl-5-nonanone (3.5%).

The location of pheromone traps is a critical factor for enhancing their efficiency [99]. For instance, Hallett et al. [140] reported that traps placed at ground level captured a significantly greater number of RPW adults than those placed at 5 m; however, the effectiveness of traps at 2 m did not differ significantly from the aforementioned heights. More recently, Al Ansi et al. [99] documented that traps positioned in shaded areas with relatively high soil moisture caught a greater number of RPW compared to traps positioned in the sun. In addition, color has been reported to affect the effectiveness of traps [119,140,141,142,143,144,145]. Hallett et al. [140] reported a higher number of RPWs were captured in black traps than in white traps, whereas Al-Saoud et al. [145] caught significantly more RPWs in red than in white or yellow traps. Additional research efforts revealed that black traps were more effective than brown, red, yellow or white traps [119,141,142].

### 5.2. Agronomic Methods

Different farming practices are imperative for managing the population of RPW, its subsequent population build-up and attack on new plantations [7,91]. In 2011, RPW infestation in Al-Ahsa Oasis were related to how farmers used three different watering methods: basin, flood, and drip. The results showed noticeable differences, with maximum (88%) RPW infestation occurring under flood irrigation followed by 9.6% and 2.4% with basin and drip irrigation, respectively [86,146]. Additionally, removing offshoot and fronds resulted in 79% weevil infestation because; 6- and 10-year-old palms were highly susceptible. In addition, the treatments for cryptic hidden breeding sites are also crucial for controlling this pest; particularly if it is found in closed gardens and other locations that are difficult to access [147]. These factors should be considered for future RPW management programs when implemented over a wide area since management on individual farms is less effective because weevils readily immigrate from neighboring fields. 

Using a novel method for the surveillance of RPW-infested palms based on street-level imagery data is a new technique for the pest’s early management [148]. The application of historical aerial photos, remote sensing images, and field surveys, integrated in a GIS environment showed that the exponential increase of RPW populations is correlated to the spatial spread model [149]. Removal of infected damaged or fallen palm trees along with trimming of dead fronts also prevents the spread of this pest by eliminating the breeding sites [6,150].

### 5.3. Biological Control

A vast array of RPW natural enemies (insects, vertebrates, and mites), and microbial control agents, entomopathogenic fungi, entomopathogenic nematodes, entomopathogenic bacteria and entomopathogenic viruses have been reported from many countries of the world [151,152]. But their implementation under field conditions has not been very successful against RPW due to its cryptic behavior. 

#### 5.3.1. Natural Enemies

The predatory and parasitic potential of insects from various orders has been well-documented against a huge variety of insect pests for centuries [151]. Several species of RPW natural enemies belong to the orders Heteroptera, Hymenoptera, Dermaptera, Coleoptera, and Diptera [152]. *Platymeris laevicollis* Distant (Hemiptera: Reduviidae) is a predator of *Oryctes rhinoceros* (L.) (Coleoptera: Scarabaeidae), which was reported to attack RPW [153]. In India, a common predator of RPW is *Chelisoches morio* (F.) (Dermaptera: Chelisochidae), which has been recorded to attack eggs and larvae of RPW in coconut tree crowns [154]. In Sicily, *Euborellia annulipes* (Lucas) (Dermaptera: Anisolabididae) was found in infested palm trees with RPW, and under laboratory conditions, demonstrated that it can predate on eggs of RPW [151,155]. Additionally in Sicily, a native parasitoid *Megascolia flavifrons* (Fabricius) (Hymenoptera: Scoliidae) has been recorded in infested palms but more studies on its biology are still needed to assess its potential as a biocontrol agent used against the different RPW life stages [151]. A few dipteran species belonging to Tachinidae and Sarcophagidae families also prey on *Rhynchophorus* spp. [152]. Lastly, *Billaea maritima* (Schiner) (Diptera: Tachinidae), a parasitoid of cetonid beetles, has been observed parasitizing RPW pupae in Sicily [156]. 

There are reports of some vertebrates (mammals and birds) feeding on RPW life stages. Two palm-dwelling mammals, *Apodemus sylvaticus* (L.) (Rodentia: Muridae) and *Rattus rattus* (L.) (Rodentia: Muridae), have been reported to eat RPW larvae, pupae and adults [151]. Concerning avian predators, *Dendrocitta vagabunda parvula* (Whistler and Kinnear) (Passeriformes: Corvidaewas) was found to prey on adults of RPW [156]. Another avian predator, *Pica pica* L. (Passeriformes: Corvidae), was reported to feed on RPW in Italy [157]. There are also reports of *Turdus merula* L. (Passeriformes: Turdidae) and *Falco tinnunculus* L. (Falconiformes: Falconidae) as potential avian predators of RPW life stages [158]. 

Mites and RPW have phoretic associations in which the mites are actively carried on the beetle body for a short period to accomplish their dispersal strategy in favorable environments [155,159,160]. These phoretic mite species belong mainly to suborder Uropodina [161,162,163,164]. So far, 25 species of mites belonging to 21 genera in 18 families have been reported to be associated with RPW [87]. Among these mites, 21 identified species (84%) belong to the order Mesostigmata followed by orders Trombidiformes (12%) and Sarcoptiformes (4%) [87]. Regarding their parasitization habit, experiments revealed that *Centrouropoda almerodai* Hiramatsu & Hirschmann (Mesostigmata: Uropodidae) reduced their host’s lifespan to 1.4 times compared to the uninfested RPWs [155]. Facultative parasitic mites *Aegyptus rhynchophorus* (Elbishlawi and Allam) and *A. alhassa* (Al-Dhafar and Al-Qahtani) (Mesostigmata: Trachyuropodidae) have been suggested as potential agents to control RPW in the field since these mites were found to feed on different developmental stages of RPW [165,166,167,168]. Apart from predation, the sheer number of phoretic mites inhabiting the RPW’s body can hinder its foraging efficiency, rendering it more vulnerable to predation [87,159]. Furthermore, a significant mite infestation can impede the host’s ability to fly and move, potentially leading to exhaustion or even death [169]. An interesting observation is the presence of more phoretic mites in male RPW than the females [87]. This fact might be attributed to male-biased association [87]. 

#### 5.3.2. Microbial Control Agents

The use of microorganisms to control insect pests is an alternative to the use of single-mode synthetic chemical insecticides. Naturally occurring biological control agents have a high degree of host specificity, low environmental toxicity, self-persistence, and minimal non-target effects. The control of RPW by using such environmentally friendly biocontrol agents would be welcomed in many date-producing regions. 

##### Entomopathogenic Fungi (EPF)

Entomopathogenic fungi play a role in regulating the population of insects in natural environments and typically pose no harm to the environment, humans, and most importantly, non-target organisms [170,171]. EPF are widespread in both forest ecosystems and agricultural settings, commercially available, and used effectively in the management of several insect pests, including RPW [172,173,174]. Extensive research has been conducted in the laboratory to assess the effectiveness of EPF against RPW [175,176,177]. Various aspects of EPF have been examined for different species, i.e. doses, strains, or application methods to determine their impact on the infection rate, field efficacy, population decrease, and mortality of RPW [58,178,179,180]. Furthermore, eggs, larvae, and adults of RPW have been assessed to determine the virulence and pathogenicity of EPF [176,177,181,182,183]. Numerous EPF species have been utilized, with different strains and isolates demonstrating notable efficacy and virulence against RPW under laboratory conditions [176,179,180,181,184]. In the field, *Beauveria bassiana* (Balsamo Crivelli) Vuillemin (Hypocreales: Cordycipitaceae) and *Metarhizium anisopliae* (Metschnikoff) Sorokin (Hypocreales: Clavicipitaceae) are commonly used in IPM programs against RPW [47,151,175,185,186,187]. For instance, *B. bassiana* significantly reduced the RPW population under field conditions [188]. In the field, *B. bassiana* and *M. anisopliae* may become an integral part of successful IPM programs targeting larvae of RPW by injecting suspensionof both fungi into infested trees targeting larvae [47,151,175,185,186,187,189]. A comprehensive list of microbial agents used for the management of RPW is provided in Table 3.

##### Entomopathogenic Nematodes (EPNs)

Over the past two decades, there has been a considerable interest in using EPNs as biological control agents against harmful insect pests [190]. EPNs, such as those from the genera *Steinernema* and *Heterorhabditis*, are effective for the management of numerous agricultural insect pests [191,192]. The third juvenile stage of EPNs, known as the infective juvenile (IJ), lives freely in the soil, harboring endosymbiotic bacteria responsible for killing their hosts. After the individual penetrates the host through natural body openings, it releases symbiotic bacteria into the host’s haemocoel. Then, the bacteria rapidly multiply and produce lethal toxins, capable of killing the host within 48 hours. Afterwards, the IJs feed on the surplus of bacterial cells, develop into adults, reproduce and when the resources are depleted, their progenies evacuate the cadaver [193]. These EPNs, along with their symbiotic bacteria are target-specific, minimizing harm to non-target organisms [193,194]. Numerous studies have evaluated the effectiveness of commonly used EPNs in both laboratory and field settings, around the Mediterranean basin, the Middle East, and southern Asia against RPW [190,194,195,196,197,198,199,200] (Table 3). In laboratory experimental efforts against RPW, several EPN species have been tested against different life stages of this pest and provided high mortality rates [194,197,199]. In most laboratory cases, *Steinernema carpocapsae* (Weiser) (Rhabditida: Steinernematidae) and *Heterorhabditis bacteriophora* Poinar (Rhabditida: Heterorhabditidae) cause high mortalities depending on the life stage of the host. Within addition to causing high mortality in RPW larvae, the use of EPNs can also cause a reduction in their feeding/foraging behavior, thus hindering growth and overall development of the adult stage. Additionally, negative effects on the fecundity of RPW adult beetles have been observed and subsequently RPW population growth is negatively affected over time [201]. In field and semi-field settings, *S, carpocapsae*, *Steinernema feltiae* (Filipjev) (Rhabditida: Steinernematidae), and *H. bacteriophora* are usually the most studied species, in regard to controlling RPW infestations [195,196,197]. Specifically, in field research settings conducted by Abbas et al. [195], larval mortality did not exceed 66,7%, among the ten local species/strains that were used. Accordingly, most of the EPN species did not manage to suppress the life stages of the RPW pest. The decrease in efficacy of EPNs can be attributed to environmental conditions, such as temperature range, as well as RPW behavior, i.e., tunneling and excessive sap production, hindering the foraging ecology of the EPNs. However, Santhi et al [198] recorded variable mortality rates in a study simulating a natural setting where the larval stages showed reduced susceptibility as their size increased, and adults or pupae were extremely susceptible to *S. carpocapsae*. One other important factor that determines the efficacy of an EPN application is the foraging behavior of IJs. For example, *S. carpocapsae* exhibited high activity in locating and infecting tunneling larvae. In order to minimize the impact of environmental constraints on EPN activity in field conditions, a chitosan adjuvant was used to protect *S. carpocapsae,* providing elevated mortality (>80%) in both preventive and curative procedures [196]. 

##### Entomopathogenic Bacteria (EPB)

EPB that have primarily been used to combat RPW belong to the families Enterobacteriaceae, Streptococcaceae, Pseudomonadaceae, and Bacillaceae [202]. The insecticidal activity of EPB originates from metabolic products that procure severe symptoms in their hosts upon infection. Notably, species from the genera *Photorhabdus*, *Xenorhabdus*, *Bacillus*, *Pseudomonas*, and *Serratia* have extensively been studied for their insecticidal properties against important agricultural pests. They infect their target and produce secondary metabolites, like enzymes and toxins that cause a variety of negative effects, including development inhibition, antifeedant behavior, and most importantly, mortality in all developmental stages [203,204,205,206,207,208,209,210,211]. In the case of RPW, Francesca et al. [212] documented the application of a highly effective *Bacillus thuringiensis* Berliner (Bacillales: Bacillaceae) strain in field trials, achieving mortality rates between 70 and 85%. The authors highlighted this strain as a key and impactful component of IPM strategies against RPW. Francesca et al. [212] efficacy finding corroborates with the results of Almasoudi et al. [213] after they tested the three isolated strains of *Serratia marcescens* Bizio (Enterobacteriales: Yersiniaceae)*, Klebsiella pneumoniae* (Schroeter) Trevisan (Enterobacteriales: Enterobacteriaceae) and *B. thuringiensis* against RPW larvae (Table 3). Interestingly, only *B. thuringiensis* provided 100% mortality [213]. Previously Pu et al. [214] reported an extension of egg hatching time, high mortality rates of *B. thuringiensis* against second and fourth instar larvae of RPW, and a reduction in the observed boring activity of treated larvae.

##### Entomopathogenic Viruses (EPVs)

The only EPV found in RPW is the highly potent cytoplasmic polyhedrosis virus (CPV). The CPV was first discovered in Kerala, India, where it infected all stages of the RPW [151]. Salama et al [215] suggested the use of this pathogen as part of a biological control strategy would not be efficient, mainly due to its low virulence. However, a recent study result exhibited a high efficacy of CPV against larvae of RPW. Specifically, a viral dose of 80 million PIB (Polyhedral Inclusion Body)/larva is highly potent, resulting in 80-100% mortality against tested larvae. Results also showed that during the larval stage, mortality generally decreased with the increase in larval developmental stage, whereas from infected pupae no adults emerged or malformed adults appeared [216].

**Table 3:** Entomopathogens used against *Rhynchophorus ferrugineus*.

Microbial Control Agent	Target Developmental Stage	Conditions	References
*Bacillus amyloliquefaciens* (ex Fukomoto) Priest et al. (Bacillales: Bacillaceae), *B. cereus* Frankland & Frankland, *B. licheniformis* (Weigmann) Chester, *B. megaterium* (de Bary) Gupta et al., *B. pumilus* Meyer and Gottheil, *B. subtilis* (Ehrenberg) Cohn, *Lysinibacillus sphaericus* (Meyer & Neide) Ahmed et al. (Bacillales: Bacillaceae)	Eggs and larvae	Laboratory	[212]
*Beauveria bassiana* (Balsamo Crivelli) Vuillemin (Hypocreales: Cordycipitaceae), *Metarhizium anisopliae* (Metchnikoff) Sorokin (Hypocreales: Clavicipitaceae)	Eggs and larvae	Laboratory	[177]
*Bacillus thurigiensis* Berliner (Bacillales: Bacillaceae)	Eggs and larvae	Laboratory	[217]
*Serratia marcescens* Bizio (Enterobacterales: Enterobacteriaceae), *Mammaliicoccus sciuri* (Kloos et al.) Madhaiyan et al. (Bacillales: Staphylococcaceae), *Klebsiella pneumonia* ssp. *pneumonia* (Schroeter) Trevisan (Enterobacterales: Enterobacteriaceae), *Proteus vulgaris* Hauser (Enterobacterales: Enterobacteriaceae), *P. mirabilis* Hauser	Larvae	Laboratory	[217]
*Steinernema carpocapsae* (Weiser) (Rhabditida: Steinernematidae)	Larvae	Laboratory	[218]
*B. bassiana*, *Cordyceps fumosorosea* Kepler, B. Shrestha & Spatafora (Hypocreales: Clavicipitaceae)	Larvae	Laboratory	[179]
*B. bassiana*	Larvae	Laboratory	[47]
*B. bassiana*	Larvae	Laboratory	[178]
*M. anisopliae*	Larvae	Laboratory	[219]
*Steinernema affine* (Bovien) Wouts, Mracek, Gerdin & Bedding (Rhabditida: Steinernematidae), *S. carpocapsae*, *S. feltiae* (Filipjev) (Rhabditida: Steinernematidae) *Heterorhabditis bacteriophora* Poinar (Rhabditida: Heterorhabditidae)	Larvae	Laboratory	[197]
*B. bassiana*	Larvae	Semi-field	[47]
*S. carpocapsae*	Larvae	Laboratory	[200]
*B. bassiana*, *M. anisopliae*, *H. bacteriophora*	Larvae	Laboratory	[174]
*B. thuringiensis*, *B. cereus*	Larvae and adults	Laboratory	[61]
*B. bassiana*	Larvae and adults	Laboratory	[220]
*B. bassiana*, *H. bacteriophora*, *B. thuringiensis* serovar *kurstaki*	Larvae and adults	Laboratory	[221]
*S. carpocapsae*, *H. bacteriophora*, *S. feltiae*	Larvae and adults	Laboratory	[190]
*Steinernema scapterisci* Nguyen & Smart (Rhabditida: Steinernematidae), *S. abbasi* Elawad, Ahmad & Reid, *S. glaseri* (Steiner) Wouts, Mracek, Gerdin & Bedding, *H. bacteriophora*	Larvae and adults	Laboratory	[199]
*S. glaseri*, *Steinernema arenarium* (Artyukhovsky) Wouts, Mracek, Gerdin & Bedding (Rhabditida: Steinernematidae), *S. carpocapsae*, *S. feltiae*, *S. riobravae* Cabanillas, Poinar & Raulston, *S. abbasi*, *S. ritteri* Doucet & Doucet, *S. kushidai* Mamiya, *Heterorhabditis* spp. (Rhabditida: Heterorhabditidae)	Larvae, pupae, and adults	Laboratory	[222]
*S. carpocapsae*	Larvae, pupae, and adults	Semi-field and field	[223]
*B. bassiana*	Eggs, larvae, and adults	Laboratory	[58]
*B. bassiana, B. brongniartii* (Sacc.) Petch (Hypocreales: Cordycipitaceae), *M. anisopliae*, *Purpureocillium lilacinum* (Thom) Luangsa-ard, Houbraken, Hywel-Jones & Samson (Hypocereales: Ophiocordycipitaceae)	Eggs, larvae, and adults	Laboratory and semi-field	[176]
*B. bassiana*	Adults	Laboratory and semi-field	[224]
*M. anisopliae*, *B. bassiana*, *Paecilomyces* sp.	Adults	Laboratory	[184]
*B. subtilis, B. thuringiensis, M. anisopliae*, *B. bassiana*, *Akanthomyces lecanii* (Zimm.) Spatafora, Kepler & B. Shrestha (Hypocreales: Cordycipitaceae)	All stages	Laboratory	[225]
*C. fumosorosea*	All stages	Laboratory and field	[226]
*A. lecanii*	All stages	Field	[227].
*S. carpocapsae*, *H. bacteriophora*	All stages	Field	[198]
*Heterorhabditis indica* Poinar, Karunakar & David (Rhabditida: Steinernematidae), *S. carpocapsae*	All stages	Laboratory	[228]
*H. bacteriophora*, *M. anisopliae*, *B. thuringiensis* serovar *kurstaki*	All stages	Laboratory and field	[229]

## 6. Effect of Temperature on Development

Among abiotic factors, temperature is a keyfactor that influences on all the developmental stages and population growth of poikilothermic organisms including insects such as RPW [230,231]. Oviposition rates and developmental stages are significantly affected by low temperatures [11,90]. For instance, the highest fecundity was recorded at 25 °C, while no egg production was observed at 15 °C when the effect of temperature which ranged between 10 and 25 °C was studied on the development of RPW under laboratory conditions [90]. In the same study, a lower temperature limit for oviposition was 15.45 °C, whereas for egg hatching it was 13.95 °C [90]. Over a wider temperature range (i.e., 21–36 °C), Peng et al. [29] reported the highest fecundity of RPW adults at 27 °C; however, there was no significant variation between temperatures that ranged from 24 to 33 °C. However, at 36 °C a significant reduction in female fecundity was observed. This relationship between temperature and fecundity ultimately affects population growth and the number of generations per year [11,232]. In areas where the mean annual temperature is lower than 15 °C, less than 1 generation per year is expected, and >2 generations can be expected when the mean annual temperature is higher than 19 °C [11].

## 7. Conclusions

Early detection of RPW infestations is critical for effective pest management, and this can be achieved through the implementation of pheromone traps. Establishing extensive monitoring systems will provide palm growers with timely warnings; thus, they will be able to take prompt actions against this noxious species. Additionally, a deep understanding of RPW biology and its interaction with host palms is essential for devising effective IPM management strategies, offering palm tree owners valuable insights into the mitigation of infestations. The implementation of plant quarantine protocols is necessary to prevent the spread of RPW populations to non-infested areas, thereby confining and mitigating the problem before it escalates. Regarding pest control, biological agents such as EPF, EPB, and EPNs have shown positive results in combating RPW populations. Continuing research into the mass production of these agents and further enhancement of their efficacy and persistence in the field could significantly improve the prospects for long-term management strategies against RPW infestations. Such strategies would prioritize environmentally friendly approaches, offering more sustainable solutions that minimize the ecological footprint of pest management methods. Overall, a multifaceted plan combining early detection, biological control, and preventive measures is imperative for the successful management of RPW infestations. The conventional management of RPW for the last twenty years has depended upon techniques like, early detection, cultural practices, biological control agents, insecticides, male-produced aggregation pheromones and mass trapping, sterile insect techniques, push–pull and attract and kill, population eradication through phytosanitation and augmentation of all management tactics [22]. However, the most recent techniques used for the detection of this pest include acoustic systems, data mining, remote sensing systems, radio telemetry, thermal and digital cameras, tree radar units (TRUs), seismic sensor-based techniques, a combination of male sterile and biocontrol agents, genomics, metabolomics, proteomics transcriptomics, volatilomics, X-ray, and microwave technology [13,22,91,233,234].

## Data Availability

Data are available within the article.
